# Subcellular localisation of *Medicago truncatula *9/13-hydroperoxide lyase reveals a new localisation pattern and activation mechanism for CYP74C enzymes

**DOI:** 10.1186/1471-2229-7-58

**Published:** 2007-11-05

**Authors:** Stefania De Domenico, Nicolas Tsesmetzis, Gian Pietro Di Sansebastiano, Richard K Hughes, Rod Casey, Angelo Santino

**Affiliations:** 1 Institute of Sciences of Food Production C.N.R. Section of Lecce, via Monteroni, 73100, Lecce, Italy; 2 John Innes Centre, Norwich Research Park, Norwich NR4 7UH, UK; 3 Dipartimento di Scienze e Tecnologie Biologiche ed Ambientali, Università del Salento, via Monteroni, 73100, Lecce, Italy

## Abstract

**Background:**

Hydroperoxide lyase (HPL) is a key enzyme in plant oxylipin metabolism that catalyses the cleavage of polyunsaturated fatty acid hydroperoxides produced by the action of lipoxygenase (LOX) to volatile aldehydes and oxo acids. The synthesis of these volatile aldehydes is rapidly induced in plant tissues upon mechanical wounding and insect or pathogen attack. Together with their direct defence role towards different pathogens, these compounds are believed to play an important role in signalling within and between plants, and in the molecular cross-talk between plants and other organisms surrounding them. We have recently described the targeting of a seed 9-HPL to microsomes and putative lipid bodies and were interested to compare the localisation patterns of both a 13-HPL and a 9/13-HPL from *Medicago truncatula*, which were known to be expressed in leaves and roots, respectively.

**Results:**

To study the subcellular localisation of plant 9/13-HPLs, a set of YFP-tagged chimeric constructs were prepared using two *M. truncatula *HPL cDNAs and the localisation of the corresponding chimeras were verified by confocal microscopy in tobacco protoplasts and leaves. Results reported here indicated a distribution of *M*.*truncatula *9/13-HPL (HPLF) between cytosol and lipid droplets (LD) whereas, as expected, *M*.*truncatula *13-HPL (HPLE) was targeted to plastids. Notably, such endocellular localisation has not yet been reported previously for any 9/13-HPL. To verify a possible physiological significance of such association, purified recombinant HPLF was used in activation experiments with purified seed lipid bodies. Our results showed that lipid bodies can fully activate HPLF.

**Conclusion:**

We provide evidence for the first CYP74C enzyme, to be targeted to cytosol and LD. We also showed by sedimentation and kinetic analyses that the association with LD or lipid bodies can result in the protein conformational changes required for full activation of the enzyme. This activation mechanism, which supports previous *in vitro *work with synthetic detergent micelle, fits well with a mechanism for regulating the rate of release of volatile aldehydes that is observed soon after wounding or tissue disruption.

## Background

Hydroperoxide lyase (HPL) is a key enzyme in plant oxylipin metabolism that catalyses the cleavage of polyunsaturated fatty acid hydroperoxides produced by the action of lipoxygenase (LOX) to volatile aldehydes and oxo acids. Depending on the substrate specificity of HPL, 6-carbon or 9-carbon aldehydes are produced from 13-hydroperoxides or 9-hydroperoxides respectively [1,2]. The synthesis of these volatile aldehydes is rapidly induced in plant tissues upon mechanical wounding and insect or pathogen attack. Together with the direct role of C9 and C6 aldehydes in defence towards different pathogens [1-3], these compounds are believed to play an important role in signalling within and between plants, and in the molecular cross-talk between plants and other organisms surrounding them [4-6]. HPL together with allene oxide synthase (AOS) and divinyl ether synthase (DES) form a cytochrome P450 (CYP) subfamily, named CYP74 (cytochrome P450, subfamily 74), specialised for the metabolism of polyunsaturated fatty acid hydroperoxides. Unlike "classical" P450 enzymes, members of the CYP74 subfamily have atypical reaction mechanisms and require neither oxygen nor a NADPH reductase. CYP74 enzymes are currently divided into four different groups on the basis of their sequence relatedness: CYP74A and B include AOS and HPL respectively, showing a strict preference for 13-hydroperoxides, CYP74C includes AOS and HPL which can convert either 9- and 13-hydroperoxides. Finally, DES are classified as CYP74D [7]. A new nomenclature for CYP74 enzymes, based upon the confirmed substrate and product specificities of recombinant proteins, has recently been proposed [8] and which assigns CYP74C to only HPLs with dual specificity.

As far as the endocellular distribution of CYP74 members is concerned, even if a plastidial localisation for AOS and HPL in CYP74A and B groups, respectively is well established, there is very little information on the subcellular localisation of plant HPLs belonging to the CYP74C subfamily. Apart from almond seed 9-HPL which is targeted to the endomembrane system and to putative lipid bodies [9], and two HPLs recently reported from rice (OsHPL1 and OsHPL2) targeted to plastids [10], there is no information about the localisation of the other HPLs in this subfamily. In contrast to almond 9-HPL which shows a strict preference for 9-hydroperoxides [9], the other members of the CYP74C subfamily can metabolise both 9- and 13-hydroperoxides and are therefore commonly referred to as 9/13-HPLs. 9/13-HPLs have been reported so far from only a few plant species, namely *M. truncatula *(Acc. No. AJ316562; [11]), melon (Acc. No. AF081955; [12]), cucumber (Acc. No. AF229811; [13]) and rice (OsHPL1, Acc. No. AK105964, OsHPL2, Acc. No. AK107161; [10]).

In the present work, we have investigated the endocellular localisation of *M. truncatula *9/13-HPL (HPLF), a member of the CYP74C subfamily and its localisation pattern was compared with that of another HPL from *M. truncatula *(HPLE) that was predicted from phylogenetic analysis [7] and confirmed through analysis of the recombinant protein (Hughes *et al*., unpublished work) to be a 13-HPL, a member of the CYP74B subfamily. The link between the unexpected localisation of a member of the CYP74C subfamily and the possible activation of the enzyme *in vivo *is therefore proposed.

## Results

### M. truncatula HPLs show different subcellular distributions

**Figure 1:**
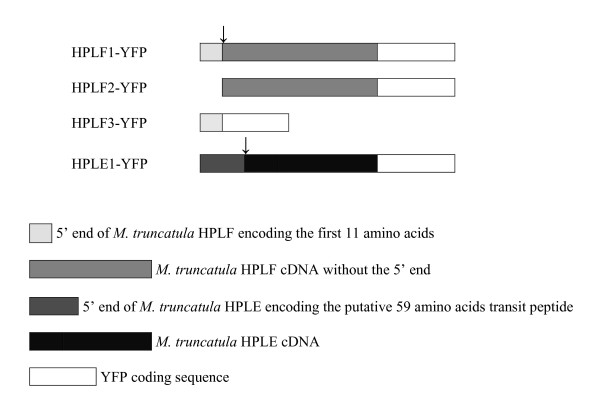
**Schematic representation of chimeric proteins used for the *in vivo *localisation of *M. truncatula *HPLs**. The arrows indicate the 11 amino acids at the N-terminus of HPLF and the 59 amino-acid transit peptide of HPLE.

Two different cDNA clones from *M. truncatula *were used in this study: the first clone (HPLF; Acc. No. AJ316562) encodes a 9/13-HPL [11] and was produced from mRNA extracted from four-week old *Rhizobium melitoti*-inoculated roots and nodules; the second clone (HPLE; Acc. No. DQ011231) encodes a 13-HPL [7] (Hughes *et al*., unpublished work) and was produced from mRNA extracted from *M. truncatula *leaves fed upon by *Spodoptera exigua *(beet armyworm) for 24 hours. Similar to other 9/13-HPLs, HPLF was not predicted to contain any canonical chloroplast transit peptide, despite having an unusual predicted N-terminal sequence enriched with serine and threonine residues (five serine residues and two threonine residues in the first eleven amino acids). Differently from HPLF, a plastidial localisation was predicted for HPLE, a putative N-terminal transit peptide of 59 amino acids was predicted by ChloroP prediction software. To study in more detail the endocellular localisation of both *M. truncatula *HPLs, a set of YFP-tagged gene fusions were prepared and the localisation of the corresponding chimeric proteins was verified by confocal microscopy after transient expression in tobacco protoplasts and leaves. Three different chimeric constructs were prepared to verify the localisation of the full length protein (pG_2_HPLF1-YFP) and the role of the first eleven amino acids at its N-terminus in the final targeting of HPLF (pG_2_HPLF2-YFP and pG_2_HPLF3-YFP). Fig. 1 shows a schematic representation of the four chimeric constructs used to investigate the localisation of HPLF and HPLE. Fluorescence patterns were monitored up to twenty four hours after transformation.

**Figure 2:**
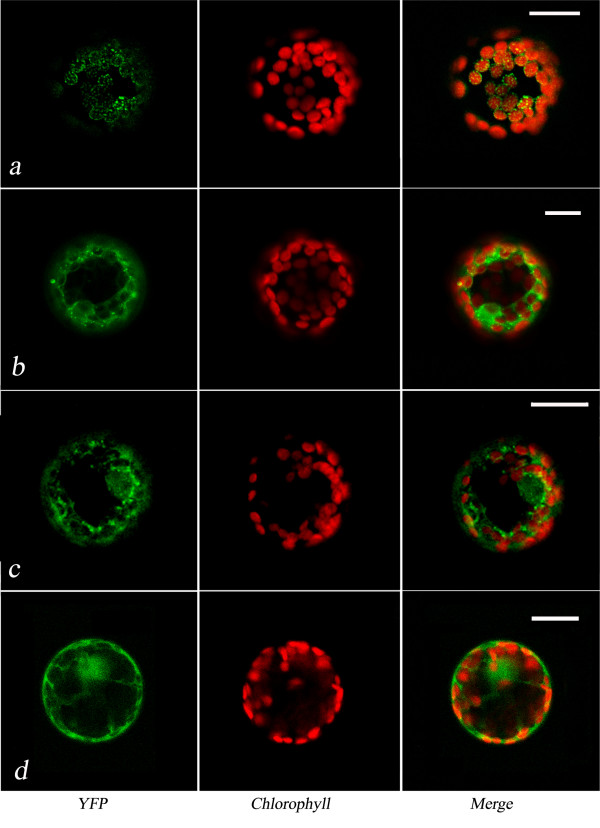
**Fluorescence patterns of representative chimeric proteins in tobacco protoplasts**. Image of a tobacco protoplast transformed with pG_2_HPLE1-YFP (**a**), pG_2_HPLF1-YFP (**b**), pG_2_HPLF2-YFP (**c**), pG_2_HPLF3-YFP (**d**) chimeric constructs. The scale bar corresponds to 20 μm.

As expected the two *M. truncatula *HPLs showed different endocellular localisations (Fig. 2). Indeed, in tobacco protoplasts expressing HPLE1-YFP, the chimera was detected as small fluorescence spots on the plastids (Fig. 2a), whereas in the case of HPLF1-YFP the fluorescence distribution was mostly cytosolic but also showed association with some small spherical bodies (Fig. 2b). A similar localisation was observed for HPLF2-YFP (Fig. 2c), whereas only a cytosolic distribution of fluorescence was found in the case of HPLF3-YFP (Fig. 2d), thus indicating that the N-terminus of HPLF does not influence the final localisation of the protein

Similar localisation results were obtained in *Nicotiana benthamiana *leaves transiently transformed with pG_2_HPLF-YFP and pG_2_HPLE-YFP chimeric constructs (data not shown).

### HPLF association with lipid droplets

**Figure 3:**
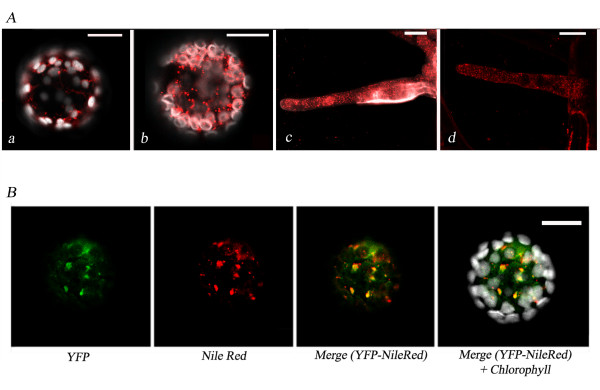
**Visualisation of lipid droplets stained by Nile red**. **A**: Tobacco and *A. thaliana *leaf protoplasts (**a**, **b**) and 80 μm confocal root projections from the same species (**c**, **d**). **B**: Image of a tobacco protoplast transformed with pG_2_HPLF1-YFP and stained with Nile red, showing several lipid droplets stained by YFP and Nile red. The scale bar corresponds to 20 μm.

When expressed in tobacco protoplasts, HPLF1/2-YFP chimeras were able to label some spherical bodies (Fig. 2) of similar size and shape to small lipid droplets (LD) which can be selectively stained in different plant tissues by Nile red, a dye which interacts with neutral lipids. Fig. 3A shows a typical visualisation of LD in tobacco and *A. thaliana *protoplasts or in *M. truncatula *and *A. thaliana *hairy-roots, selectively stained by Nile red. Co-localisation of YFP and Nile red fluorescences was also verified in tobacco protoplasts expressing the HPLF-YFP chimera (Fig. 3B).

**Figure 4:**
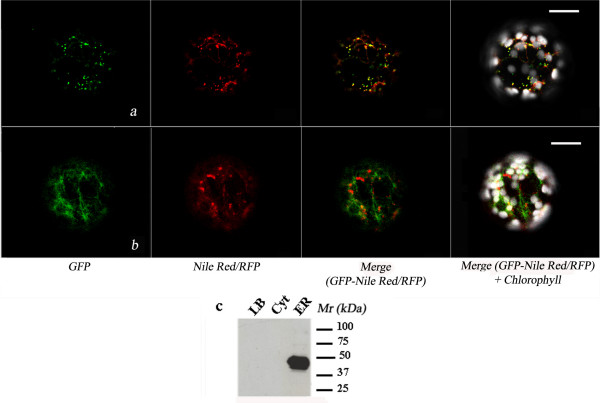
**Localisation of OLE-GFP/RFP in tobacco protoplasts**. (**a**): Image of a tobacco protoplast transformed with OLE-GFP and stained with Nile red. (**b**): Image of a tobacco protoplast co-expressing GFP-KDEL and OLE-RFP. The scale bar corresponds to 20 μm. (**c**): The lipid bodies (LB), ER and cytosolic (Cyt) protein fractions recovered from tobacco protoplasts transformed with OLE-GFP were subjected to SDS-PAGE and Western blot analysis using a GFP antiserum.

To verify if LD could be also the main destination of ectopically expressed oleosin, tobacco protoplasts were transformed with oleosin-GFP chimeric construct and stained with Nile red. As shown in Fig. 4a, the two fluorescences showed a prevalent co-localisation, even if in some cases, some spots were labelled only by GFP fluorescence or Nile red staining. These data could reflect the fact that LD are already pre-formed in tobacco protoplasts (as already shown in Fig. 3A) and that, some of newly synthesised oleosins are not yet incorporated in LD.

To better study the relationship between LD and the ER, oleosin-RFP (OLE-RFP) was co-expressed together with GFP-KDEL (to label the ER) in tobacco protoplasts. Our results (Fig. 4b) indicated that oleosin-RFP is rapidly sorted to LD which in some cases (see the large red spots of Fig. 4b) appeared to be labelled by RFP alone. Considering that LD were very close to the ER, it was very difficult to discriminate exactly about the relationship that existed between them. Finally, we isolated lipid bodies, microsomal and cytosolic protein fractions from tobacco protoplasts expressing oleosin-GFP and carried out western-blot analysis using an anti-GFP antibody. As shown in Fig. 4c, oleosin-GFP was detected in the ER fraction, thus indicating that, in our experimental conditions, LD are recovered in such a fraction. A faint band of the molecular mass predicted for oleosin-GFP was also found in the lipid body fraction at longer exposure (data not shown). This observation supports the hypothesis that, in our experimental conditions, LD are recovered mostly from the ER fraction.

**Figure 5:**
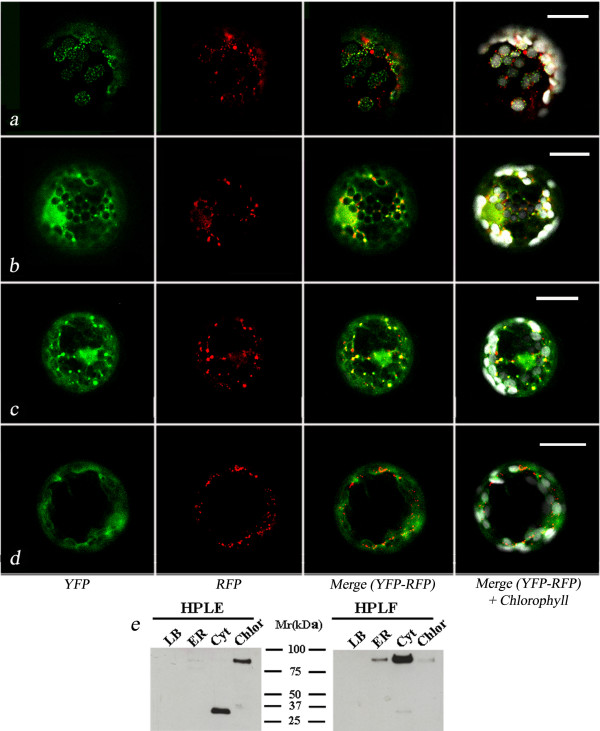
**Representative image of HPLE/F-YFP fluorescence distribution in the presence of oleosin**. (**a**): Tobacco protoplasts co-expressing pG_2_HPLE1-YFP and OLE-RFP. (**b**): Tobacco protoplasts co-expressing pG_2_HPLF1-YFP and OLE-RFP. (**c**): Tobacco protoplasts co-expressing pG_2_HPLF2-YFP and OLE-RFP. (**d**): Tobacco protoplasts co-expressing pG_2_HPLF3-YFP and OLE-RFP. The scale bar corresponds to 20 μm. YFP (505–530 nm) fluorescence in green. (**e**): The lipid bodies (LB), ER and cytosolic (Cyt), chloroplastid (Chlor) protein fractions recovered from tobacco protoplasts co-expressing OLE-RFP and HPLE/F-YFP were subjected to SDS-PAGE and Western blot analysis using a GFP antiserum.

With the aim to better study the association of HPLF with LD, we carried out co-expression of YFP-tagged *M. truncatula *HPLs and oleosin-RFP chimeric constructs in tobacco protoplasts. As shown in Fig. 5b–c, HPLF1/2-YFP chimeras showed a prevalent, even though not complete, co-localisation with oleosin-RFP fluorescence in LD. Co-expression of OLE-RFP and HPLF3-YFP chimeras did not succeed in targeting YPF to LD, which were only labelled by RFP (Fig. 5d).

Finally, in tobacco protoplasts co-expressing OLE-RFP and HPLE1-YFP, YFP fluorescence was detected on the plastids as small spots similar to those reported in Fig. 2a and was physically separated by RFP fluorescence (Fig. 5a). However, in some cases LD, labelled by oleosin RFP, were very close to plastids and RFP and YFP fluorescences seemed to co-localise. The physiological significance of such an association is currently unclear and further experiments are in progress to clarify it.

To confirm the confocal microscopy results, we carried out sub cellular fractionation of tobacco protoplasts co-expressing OLE-RFP and HPLE/F-YFP. Plastidial, microsomal, lipid bodies and cytosolic protein fractions were isolated as described in the *Materials *section. As shown in Fig. 5e, the full chimera of HPLE1-YFP was detected only in the plastidial fraction. The lower molecular weight polypeptide immunodetected in the soluble protein sample may be due to some proteolytic degradation of the chimera which produces a soluble polypeptide. Since no cytosolic distribution of fluorescence was observed in confocal images, it appeared evident that this fragment was unable to fold correctly and be fluorescent.

**Figure 6:**
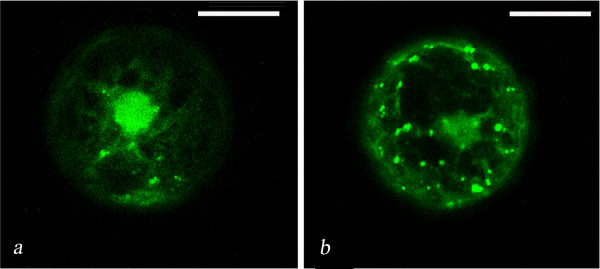
**Representative image of HPLF-YFP fluorescence distribution in the presence and absence of oleosin**. Tobacco protoplasts expressing HPLF-YFP (**a**) or co-expressing HPLF-YFP and oleosin-RFP (**b**). Images are 1.6 μm confocal images, YFP (505–530 nm) fluorescence in green.

HPLF1-YFP was mainly found in the cytosolic protein fraction, even though a clear band was also detected in the microsomal fraction, thus confirming the localisation of HPLF1 with ER associated LD. A faint band was also detected in the plastid fraction. These results could be indicative of a limited interaction of HPLF with the outer membrane of this organelle. In this context, confocal images showed that, in some cases, YFP fluorescence was very close to plastids (Figs. 2 and 5). Confocal images also showed a nuclear localisation for HPLF-YFP (Figs. 2, 5, 6). This pattern was interpreted as a sign of solubility of the chimera in the cytosol. Despite the large size of HPLF-YFP, the negligible amount of degraded YFP detected in western blot (see Fig. 5e) confirmed this interpretation.

Interestingly, in tobacco protoplasts co-expressing OLE-RFP and HPLF1/2-YFP chimeric proteins, the amount of YFP fluorescence associated with LD showed a significant increase (compare Figs. 2 and 5). A precise quantification of this change in fluorescence distribution appeared difficult since each cell can express a different amount of protein within the same population. Therefore, we counted the LD detected in several tobacco protoplasts expressing HPLF1/2-YFP or co-expressing HPLF1/2-YFP and oleosin-RFP. In the protoplasts expressing both the chimeric proteins the number of LD detected was three/four times greater than that found in protoplasts expressing HPLF-YFP alone. A representative image of HPLF1-YFP fluorescence distribution in the presence and absence of oleosin is shown in Fig. 6.

### Purified seed lipid bodies can activate HPLF

**Figure 7:**
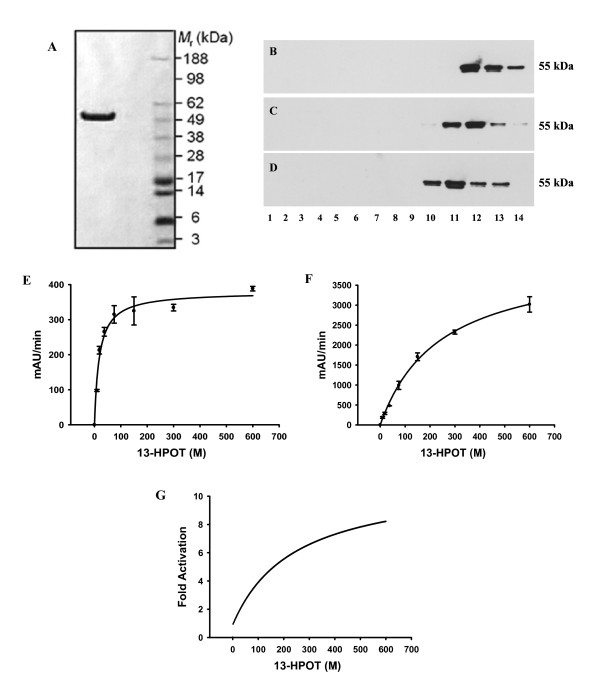
**Effect of detergent or lipid bodies on the enzymatic activity of HPLF**. (**A**): SDS-PAGE gel electrophoresis of HPLF after purification by IMAC; (**B**-**D**): Sedimentation analyses. Purified HPLF was incubated in the presence of purified seed lipid bodies (**B**), 5 mM Emulphogene detergent (**C**) or 100 mM sodium phosphate buffer, pH 6.5 (**D**) and loaded onto linear 5–20% sucrose gradients. After centrifugation the gradients were fractionated and analysed by SDS-PAGE and Western-blot analyses using a specific His-tag antiserum. Numbering refers to the 14 fractions collected from the bottom of the gradients. (**E**, **F**): Kinetic analysis of HPLF in the presence and absence of lipid bodies. HPLF (1.8 pmol) diluted in 100 mM sodium phosphate buffer, pH 6.5 alone (**E**), or in the same buffer containing 0.3 M sucrose and lipid bodies (**F**) was assayed with 13-HPOT (0–640 μM) under the standard assay conditions (See Materials and Methods). (**G**): Fold-activation of HPLF by lipid bodies as a function of 13-HPOT concentration. Fold-activation is defined as the ratio of the activity in the presence of lipid bodies/activity in the absence of lipid bodies, determined from the kinetic plots in (**E**) and (**F**).

In a previous work [11], we showed that recombinant HPLF purified to homogeneity from *E. coli *cultures is active in the absence of detergent. Nevertheless, the specific activity of the detergent-free protein is greatly reduced in comparison with the activity recorded with the enzyme solubilised in a detergent-containing buffer, or after treatment of the detergent-free protein with detergent micelles. To verify if purified seed lipid bodies could induce the conformational changes required for HPLF activation, the enzyme was purified to homogeneity by immobilised metal affinity chromatography (Fig. 7A). Sedimentation analyses on linear sucrose gradients were than compared of the native detergent-free HPLF with the same enzyme solubilised in the presence of seed lipid bodies (purified by sequential washing steps without any detergent) or 5 mM Emulphogene. As shown in Fig. 7B and 7C, HPLF solubilised in the presence of lipid bodies or detergent peaked at the same fractions (about 8% sucrose concentration), thus showing the same sedimentation constant. In contrast, the native detergent-free HPLF showed a different sedimentation constant (it peaked one fraction earlier, about 8.4% sucrose concentration; Fig. 7D). Furthermore, the different fractions recovered from sucrose gradients after HPLF solubilisation in the presence of lipid bodies, were separated by SDS-PAGE and stained by Coomassie blue (data not shown). Our results indicated that oleosin and HPLF peaked at the same fractions, thus confirming the association between HPLF and lipid bodies.

Finally, we determined the *K*_m _and *k*_cat _of purified HPLF with 13-HPOT, the preferred substrate of the enzyme, in the presence and absence of purified lipid bodies. A comparison of Figs. 7F and 7F shows clearly that the kinetics of the interaction between the preferred substrate 13-HPOT and HPLF is dramatically affected by the presence of lipid bodies. The *k*_cat _was increased 11-fold in the presence of lipid bodies, which was very similar to the fold-increase observed using synthetic detergent micelle [11]; the *k*_cat _value of 724 s^-1 ^indicates that HPLF was fully activated by lipid bodies. Despite the fact that the overall *k*_cat_/*K*_m _ratio is relatively unchanged after binding to lipid bodies, it is clear from a plot of substrate concentration vs fold activation (Fig. 7E, calculated as the ratio of activity with lipid bodies/activity without lipid bodies using the fitted data in Figs. 7C, 7D) that HPLF is increasingly activated in response to substrate supply, and would almost certainly be activated at physiologically relevant concentrations. This demonstrates unambiguously that HPLF was activated in the presence of lipid bodies.

## Discussion

Volatile aldehydes, produced by the action of HPL are an essential component of plant oxylipin metabolism, and play an important role in the plant-environment interaction [4-6]. Most results obtained to date refer to members of the CYP74B subfamily, which includes 13-HPLs that are expressed in aerial tissues and associated with plastids. In the case of HPLE, we have similarly shown that the full length sequence was able to route YFP to plastids in transiently transformed tobacco protoplasts and leaves. The fluorescence patterns observed during transient expression of HPLE1-YFP were similar to those recently reported for potato HPL and AOS enzymes, where the corresponding GFP-tagged chimeras resulted in fluorescent dots associated with thylakoid membranes [14]. Further experiments are in progress to verify if *M. truncatula *HPLE can share a similar localisation inside the plastids.

We have presented new data on the subcellular distribution of 9/13-HPLs belonging to the CYP74C subfamily. 9/13-HPLs were initially thought to be restricted to the *Cucurbitaceae *family, but their occurrence in other plant species, such as *Medicago *spp. and rice have been reported only recently [10,11]. Transient expression in tobacco protoplasts and leaves, of YFP-tagged HPLF enabled us to carry out a detailed localisation of this enzyme. Our results indicated that a cytosolic distribution of fluorescence co-exists with the fluorescence associated with small spherical organelles.

In previous work [9] we showed that another member of the CYP74C sub-family, a 9-HPL from almond seed, associates with similar organelles even though it was mainly localised in the microsomes. In this context, the localisation pattern of the almond 9-HPL differs significantly from the cytosolic distribution of HPLF and this is the first report showing such a localisation for HPL.

In the present work, we first showed, by co-localisation experiments either with oleosin-GFP/Nile red and oleosin RFP/GFP-KDEL (shown in Fig. 4), that oleosins, when ectopically expressed in tobacco protoplasts, are specifically targeted to lipid droplets (LD). LD consist of a core of neutral lipids surrounded by a surface monolayer of phospholipids and form from specific ER sub-compartments, where neutral lipids are synthesised and accumulated [for a review see [15] and [16]]. Western blot analyses indicated a main microsomal localisation for oleosin, when expressed in tobacco protoplasts (Fig. 4c). Together with the confocal images shown in Fig. 4a and 4b, these results could indicate that, in such a system, LD are mainly connected to the ER. A support to this interpretation may come from studies in animals, where they have been extensively studied as a fundamental components of intracellular lipid homeostasis [16]. A prevalent ER localisation was recently reported for adipophilin one of the main LD-associated proteins in animal cell [17]. In this study it was also reported the association of adipophilin with the cytoplasmic leaflet of ER, closely apposed to the LD envelope, Noteworthy, they demonstrated for the first time that LD is not situated within the ER membrane; but rather both ER membranes follow the contour and enclose LD. If such ER localisation can be shared by oleosin, when expressed in leaves, still awaits to be confirmed.

The presence of LD showing different size and features cannon be excluded from results reported in Fig. 4. Indeed in some cases Nile red and oleosin-GFP do not co-localise and some LD appeared labelled by only one fluorescence. Moreover, the size of several LD increased significantly in the presence of oleosins. The presence of LD of different size was already reported by Liu et co-workers [18]. They reported a different localisation for a GFP-tagged barley caleosin (HvClo1-GFP) and RFP-tagged oleosin (HvOle-RFP) in leaf epidermal cells after six hours post-transformation. Indeed, HvClo1-GFP was initially associated with small lipid droplet, whereas oleosin-RFP associated with bigger *bona fide *lipid bodies. Interestingly, the size of these lipid bodies increased with time together with the co-localisation between the two proteins.

Our results also indicated that *M. truncatula *HPLF specifically interacts with LD. In this context, co-localisation experiments with Nile red/oleosin-RFP and HPLF-YFP were further confirmed by western-blot analyses showing that HPLF was also detected in the ER fraction, where LD are recovered, together with the cytosolic fraction (Fig. 5).

The cytosolic distribution of HPLF-YFP was characterised by the labelling of the nucleus. Such a nuclear localisation was unexpected because of the large size of chimera. Anyway, it was certainly due to the full chimera since no significant degradation products were detected by western blot analysis (Fig. 5e).

Interestingly, our results indicated that the amount of HPLF associated with lipid bodies increased in the presence of oleosin (Fig. 6). The interpretation of images in this sense was supported by the observation that in all images analysed, the number of LD significantly increased in the presence of OLE-RFP.

A key role has been proposed for LD in re-mobilisation of membrane lipids during senescence of some, an possibly all, plant tissues [19]. Results here presented together with others [9,20] pointed out the specific association with LD of enzymes, i.e. HPL and peroxygenase, involved in plant lipid metabolism and oxylipins biosynthesis.

At present the factors governing the association of HPLF with LD are unclear. However, it is possible to hypothesise a peripheral interaction between the phospholipid monolayer of LD and a hydrophobic feature displayed on the surface of the HPLF protein.

The HPLF cDNA clone was isolated from mRNA extracted from four-week old *R. melitoti*-inoculated roots and nodules. Notably, several LD were labelled with Nile red in *M. truncatula *and *A. thaliana *hairy roots, thus demonstrating the presence *in vivo *of lipid storage compartments in this non-oil storing tissue where HPLF is expressed (Fig. 3A). The molecular organisation of root LD is still debated and currently it is unclear if they can share a similar organisation with seed lipid bodies. In the roots of *A. thaliana *plants expressing a sunflower oleosin, the protein was detected in the ER but not in the lipid body fraction [21]. However, in rapeseed root tips, it was reported that both caleosin and oleosin were detected, by immunoblotting and immunolocalisation analyses, in the lipid body fraction [22].

The kinetic analyses we carried out on purified HPLF, clearly indicated that the interaction with substrate is dramatically affected by the presence of purified lipid bodies. The increase (11-fold) in the *k*_cat _observed in the presence of lipid bodies was very similar to the fold-increase observed using synthetic detergent micelle [11] and demonstrates unambiguously that HPLF was fully activated in the presence of lipid bodies. Unexpectedly, this increase in *k*_cat _was associated with a 13-fold reduction in substrate affinity, which was opposite to that observed with synthetic detergent micelle. This probably reflects differences in HPLF binding to the smaller, more defined, detergent micelles which is presumably much tighter than binding to the larger, more irregular lipid bodies. Nevertheless, the looser binding to lipid bodies is clearly sufficient to promote the changes in protein conformation required to induce the rapid increases in substrate turnover.

Future studies will hopefully be directed at examining the effects of other purified membrane fractions, on CYP74 enzyme activation.

## Conclusion

**Figure 8:**
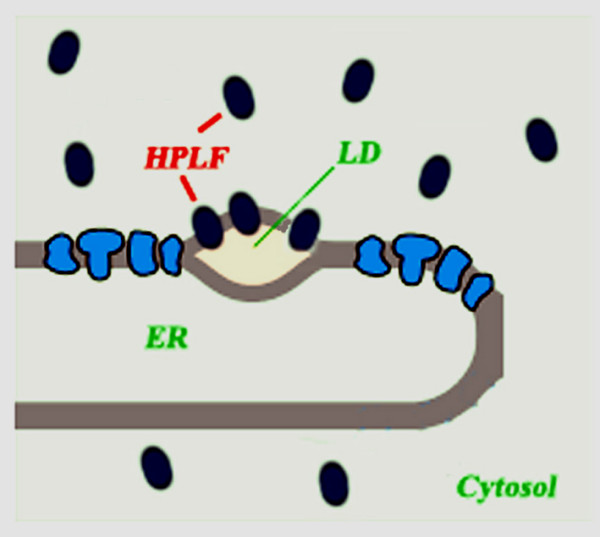
**Schematic representation of *M. truncatula *HPLF localisation in transiently transformed tobacco protoplasts**. The protein was targeted to cytosol and lipid droplets (LD).

We provide evidence for the first CYP74C enzyme, to be targeted to the cytosol and lipid droplets (a schematic representation is shown in Fig. 8). We have also showed by sedimentation and kinetic analyses carried out on purified HPLF, that the association with LD or lipid bodies can result in the protein conformational changes required to fully activate the enzyme. This activation mechanism, which supports previous *in vitro *work with synthetic detergent micelle, fits well with a mechanism for regulating the rate of release of volatile aldehydes that is observed soon after wounding or tissue disruption. Further work is needed to identify the molecular mechanisms governing the distribution of HPLF inside the cell.

## Methods

### Gene constructs and vector mobilization

HPLE (Acc. No. DQ011231) was tagged with YFP by directional cloning to the 5' end of the enhanced YFP (EYFP) gene (Clontech) through the *Asc*I, *Not*I restriction sites. The restriction sites were inserted in the HPLE sequence using the following primers: 5'-TAGGCGCGCCATGTCACTCCCACCACCGATACC-3' (forward) and 5'-TGCGGCCGCCTTTGGCCTTCCTTAAGGCAGTAATGG-3' (reverse). The amplified product was cloned into a modified pGreenII0029 plant expression vector [23] upstream of the YFP coding sequence. Expression was driven by a double 35S promoter and 35S terminator. The final construct was named pG_2_HPLE1-YFP.

Various fragments of HPLF (Accession No. DQ011231) were tagged with YFP using the same restriction sites and the same expression vector as that used for HPLE. Restriction sites were inserted into the *M. truncatula *HPLF cDNA using the following forward primers: 5'-TAGGCGCGCCATGGCTTCCTCATCAGAAACCTCC-3' for pG_2_HPLF1-YFP construct and 5'-TAGGCGCGCCATGCTCCCCTTGAAACCAATCCCAG-3' for pG_2_HPLF2-YFP construct and a common reverse primer: 5'-TGCGGCCGCCGACGGTGGATGAAGCCTTAACAAGTG-3'. The 5' end of *M. truncatula *HPLF encoding the first 11 amino acids was tagged with YFP using the following two primers: 5'-CGCGCCATGGCTTCCTCATCAGAAACCTCCTCAACCAACGGC-3' (forward) and 5'-GGCCGCCGTTGGTTGAGGAGGTTTCTGATGAGGAAGCCATGG-3' (reverse) to obtain the chimeric construct named pG_2_HPLF3-YFP. The cross-dimer produced was subsequently cloned into the expression vector digested with *AscI *and *NotI*.

Oleosin-GFP (OLE-GFP) construct was obtained as above reported [9]. Oleosin-RFP (OLE-RFP) construct was obtained replacing GFP with RFP (kindly provided by Dr. Tsien) using the following primers: RFPNhe (5'-AAA GCT AGC ATG GCC TCC TCC GAG GAC GTC- 3') was used to insert the *Nhe*I site and the reverse primer RFPSph (5'-AAA GCA TGC TTA GGC GCC GGT GGA GTG GCG- 3') was used to insert the *Sph*I site.

The GFP-KDEL chimeric construct was prepared as described [24]. Expression was driven by the 35S promoter and *nos *terminator.

### Plant cultivation and protoplast transient expression

Seeds of *Nicotiana tabacum *(cv. SR1), *A. thaliana *(ecotype Columbia), *M. truncatula *were germinated and grown in sterile conditions on solid Murashige and Skoog (MS) medium supplemented with 3% sucrose at 26°C under continuous illumination. For root observation and easy removal before staining, seedlings were grown on vertically oriented MS plates for 5 days [25]. Tobacco and *A. thaliana *protoplasts were isolated as previously reported [26], then cultured and rinsed using the indicated media and transformed by PEG-mediated direct gene transfer essentially as described [27,28]. Ten micrograms of plasmid were used for the transformation of about 600000 tobacco protoplasts. Two hours after addition of PEG and plasmid DNA, the protoplasts were rinsed to remove the PEG, resuspended in 2 ml culture medium and incubated at 26°C in the dark.

### Lipid staining

Protoplast staining with Nile red was carried out as reported [9], with the only exception that Nile red was used instead of Nile blue. Protoplasts were observed after 10 min. incubation in protoplast medium supplemented with 1 mg/ml dye solution, without any washing step. For root staining, *A. thaliana *roots were incubated in a solution of 1 mg/ml Nile red for 5 min., washed with sterile water and observed by confocal microscopy.

### Confocal laser scanning microscopy

Protoplasts transiently expressing fluorescent constructs were observed by fluorescence microscopy in their culture medium at different times after transformation. They were examined with a confocal laser-microscope (LSM Pascal Zeiss). GFP and YFP were detected with the filter set for FITC (505–530 nm), RFP with a 560–615 nm filter set, while chlorophyll epifluorescence was detected with the filter set for TRITC (> 650 nm). An excitation wavelength of 488 nm was used. To detect Nile red fluorescence, an excitation wavelength of 488 nm was used and the emission was recorded with the 560–615 nm filter set. The "profile" function of Zeiss Pascal software was used to estimate the YFP fluorescence in adjacent areas/lines of the same cell. Fluorescence in lipid bodies labelled by HPLF1/2-YFP was always stronger than in other unidentified areas/structures. The ratio between these fluorescence values was calculated in the presence and absence of OLE-RFP chimera and led us to appreciate a 3–4 fold increase in all analysed images when OLE-RFP was co-expressed.

### Protoplast fractionation

Protoplast pellets (6 × 10^6 ^cells) were resuspended in 5 ml sucrose buffer (0.5 M sucrose in 150 mM Tris-HCl pH 7.5, 1 mM EDTA, 10 mM KCl, 1 mM MgCl_2_, 2 mM DTT) supplemented with protease inhibitors (Sigma) and lysed by three consecutive freezing-thawing cycles. Intact cells and debris were removed by centrifugation for 5 min at 500 × *g*. The supernatant was centrifuged again at 5000 × *g *to separate the crude plastidial fraction (fraction A) from the other proteins (fraction B). The fraction A was resuspended in sucrose buffer and layered onto a three steps sucrose gradient consisting of 1.45, 0.84, 0.45 M sucrose and centrifuged at 100,000 × g for 1 hr at 4°C as previously described [29]. After centrifugation intact plastids were recovered at the interface between 1.45 and 0.80 M sucrose, diluted with 100 mM Tris-HCl, pH 8.0 and centrifuged again at 10000 × g for 10 min at 4°C. The pellet (plastid fraction) was resuspended in SDS-PAGE sample buffer.

Fraction B was used to separate lipid bodies (LB), microsomes and cytosol fractions by two-layer flotation as above described [9]. After centrifugation at 100000 × *g *for 1 h at 4°C, the following fractions were recovered: the LB fraction from the top of the gradient; the cytosolic protein fraction (10000 × *g *supernatant); and the microsomal fraction (100000 × *g *pellet). The pellet (microsomes) was resuspended in SDS-PAGE sample buffer, whereas the proteins from the LB and cytosolic protein fraction were precipitated with trichloroacetic acid and resuspended in SDS-PAGE sample buffer.

### HPLF purification, kinetic analyses and purification of seed lipid bodies

Recombinant HPLF was purified to homogeneity from *E. coli *BL21 (DE3) cells by immobilised metal affinity chromatography (IMAC) as described previously [11]. Steady state kinetic data were collected using Shimadzu kinetics software (version 2.7). Activity was determined at 25°C in a standard assay containing 100 mM sodium phosphate buffer, pH 6.5 by monitoring the disappearance of substrate at 234 nm. Substrate was diluted from a 20 mM stock that was stored at -70°C in ethanol; the exact concentration after dilution was determined using a molar extinction of 25 mM^-1^.cm^-1^. *K*_m _and *k*_cat _for 13-HPOT were calculated by fitting the data sets to a one site saturation model for simple ligand binding using SigmaPlot 8 (Sigma-Aldrich). Lipid bodies were isolated from water melon seeds, by two-layer flotation as previously reported [9], further purified by two sequential washings with 2.0 M NaCl and finally resuspended in 150 mM Tris-HCl, pH 7.5, containing 0.6 M sucrose.

### Rate zonal sucrose gradients

Different aliquots of purified HPLF (about 10 μg) were incubated with 100 mM sodium-phosphate buffer pH 6.5, purified lipid bodies or 5 mM Emulphogene for 15 min at 25°C and than loaded onto linear 5 to 20% (w/w) sucrose gradients (in 20 mM Tris-HCl pH 7.5, 100 mM NaCl) and centrifuged at 150000 × g for 20 h. After centrifugation, 1 ml fractions were collected from the bottom of the tube and the sucrose concentration determined. Proteins from each aliquot were precipitated with trichloroacetic acid and resuspended in SDS-PAGE sample buffer. Western blot analyses were performed according to the ECL protocol (Amersham) and a 1:4000 dilution of an anti-His antiserum (Sigma).

### SDS/PAGE and Western blot analysis

Proteins were subjected to SDS-PAGE and transferred to nitrocellulose membrane (Amersham). Western blot analyses were performed according to the ECL protocol (Amersham) and a 1:10000 dilution of an anti-GFP antiserum (Sigma).

## Abbreviations

CYP74, Cytochrome P450 subfamily 74; Emulphogene, polyoxyethylene 10 tridecyl ether; 9-HPOD, 9(*S*)-hydroperoxy-(10*E*, 12*Z*)-octadecadienoic acid; 9-HPOT, 9(*S*)-hydroperoxy-(10E, 12*Z*, 15*Z*)-octadecatrienoic acid, 13-HPOD, 13(*S*)-hydroperoxy-(9*Z*, 11*E*)-octadecadienoic acid; 13-HPOT, 13(*S*)-hydroperoxy-(9*Z*, 11*E*, 15*Z*)-octadecatrienoic acid; HPL, hydroperoxide lyase; HPLF, *M. truncatula *9/13-HPL; HPLE, *M. truncatula *13-HPL; P450, cytochrome P450.

## Authors' contributions

SDD carried out confocal analyses on tobacco protoplasts and prepared some chimeric constructs used in this work; NT prepared HPL-YFP chimeric constructs and carried out confocal analyses on transiently transformed *N. benthamiana *leaves; GPDS made the microscopic observations; RKH purified HPLF and carried out the kinetic analysis of HPLF in the presence and absence of lipid bodies, AS carried out protoplasts fractionation, Western-blot analyses, sedimentation analyses in the presence of purified lipid bodies. AS together with RC and RKH edited the final manuscript. The authors read and approved the final manuscript.
